# Obesity Patterns among Women in a Slum Area in Brazil

**DOI:** 10.3329/jhpn.v29i3.7876

**Published:** 2011-06

**Authors:** João G. Alves, Romero W. Falcão, Renato A. Pinto, Jailson B. Correia

**Affiliations:** ^1^Department of Epidemiology, Instituto de Medicina Integral Prof. Fernando Figueira, Rua dos Coelhos, 300-Boa Vista, Recife (PE), Brazil; ^2^Escola Pernambucana de Saúde, Recife, Brazil

**Keywords:** Caloric intake, Cross-sectional studies, Obesity, Physical activity, Slum, Brazil

## Abstract

High-energy diet and sedentary lifestyle fail to completely explain the epidemic of obesity in developing countries. In this cross-sectional survey, the prevalence and patterns of overweight/obesity were assessed among women in a slum in Brazil. Using anthropometric measurements, shorter form of the International Physical Activity Questionnaire (IPAQ), and a 24-hour diet recall questionnaire, data were collected from 632 women aged 20-60 years. The prevalence of overweight and obesity was 29% and 17% respectively. Physical inactivity was found in 17% of the women; 12% of them had short stature, and 44% had energy intake below the recommended dietary allowance. Results of multiple logistic regression showed that overweight/obesity differed significantly (p<0.05) in the following aspects: abdominal circumference, energy intake, and short stature. A high prevalence of overweight/obesity was found in a very poor community associated with high-energy intake and short stature.

## INTRODUCTION

Obesity and obesity-related morbidity are serious public-health problems in developing countries (1,2). While high-energy diet and sedentary lifestyle have been described as primary factors for the worldwide epidemic of obesity (3,4), these features fail to completely explain the epidemic of obesity in developing countries (5,6).

According to the thrifty phenotype hypothesis, nutritional deprivation early in life gives rise to adaptive mechanisms that could result in greater susceptibility to obesity in adult life (7). Chronic undernutrition in early life also results in linear growth reduction (8,9). Furthermore, short stature, an indicator of previous undernutrition, has been associated with obesity in some studies (10,11).

In Brazil, nearly 50 million people live in slums or *favelas.* These heavily-populated urban areas are characterized by poverty and poor housing (12). People from the *favelas* often share a history of migration from the countryside to the city and the past precarious socioeconomic conditions associated with a high burden of malnutrition in early life. On the other hand, life in the slum leads to changes in diet and physical activity. Therefore, this population provides conditions to test the role of energy intake, physical activity, and previous malnutrition in the genesis of obesity. This study describes the prevalence and patterns of overweight/obesity, specifically energy intake, physical activity, and height, among slum women.

## MATERIALS AND METHODS

A cross-sectional survey was carried out during April-October 2009 in Santo Amaro-1, an inner city slum in Recife, northeast Brazil. Santo Amaro-1 occupies an area of about one sq km and is one of the three neighbouring slums located about 5 km north of Recife commercial centre. It has an estimated population of 3,340, of whom 2,256 are registered with the government Family Health Programme (FHP). The target population of this study included 752 women, aged 20-60 years, who were registered with the FHP at the time of enrollment. Thirty-six women were excluded due to physical handicap or pregnancy at the time of enrollment.

Weight was assessed using electronic scales with a capacity of 150 kg and precision to the nearest 100 g (Filizola®). Height was measured using a stadiometer with a non-extensible two-metre measuring tape accurate to the nearest 0.1 cm. Body mass index (BMI) was given by the quotient of body-weight (kg) and the square of the height (m^2^). Overweight and obesity were determined by BMI as ≥25 kg/m^2^ and ≥30 kg/m^2^ respectively. Women were considered to be of short stature if their height was equal to or lower than the 5th percentile of the height distribution of the Brazilian population of the same gender (≤149 cm). Waist-circumference was measured using a non-extendable tape graduated in 0.1-cm divisions, at a level midway between the iliac crest and the lower anterior superior rib.

Following training, community health workers administered the shorter version of the International Physical Activity Questionnaire (IPAQ). The validi-ty and reliability of the IPAQ has been tested in several settings, including a Brazilan population (13). Standard scoring of the IPAQ interpretation was used (http://www.ipaq.ki.se/scoring.pdf).

The community health workers also administered the 24-hour diet recall questionnaire in a weekday. Subjects were asked what they had eaten in the previous day. For each food item, subjects identified the serving-size (small, medium, or large) and frequency of consumption. To help subjects estimate portion-sizes, food pictures were used. Energy intake was calculated using the Brazilian food-composition tables (14).

Analyses were performed using the SPSS software (version 12.0) (SPSS Inc., Chicago, IL). The significance level adopted was a p value of ≤0.05. Data are presented as means, standard deviation, and ranges. Student's non-paired *t*-test was used for comparing pairs of means. Variables showing a p value of <0.20 in bivariate analysis were included in multiple regression analysis to assess association with overweight/obesity.

The Research Ethics Committee of the Instituto de Medicina Integral Prof. Fernando Figueira approved the study (protocol number 18308), and the participants gave written consent.

## RESULTS

Of the 716 women eligible for the study, 632 (88.2%) completed assessment. Their mean age was 42.6±16 years; 41% were married; 40% had mixed colour; 82% had a religion affiliation; and 75% were unemployed. Reasons for non-participation of 84 women included absence from home in the three diurnal home-visits (n=23), refusal (n=32), inability to locate (n=16), and other reasons (n=13).

The prevalence of overweight and obesity was 29% and 17% respectively. Physical inactivity was found in 17% of the women; 12% had short stature; and 44% had energy intake below the recommended dietary allowance. Results of bivariate analysis showed that overweight/obesity differed significantly (p<0·05) in the following aspects: (a) short stature, (b) abdominal wasting, and (c) energy intake (Table 1). The same variables remained statistically significant after adjusting in a multiple logistic regression model (Table 2).

**Table 1.** Women with overweight/obesity according to demographic and socioeconomic variables

Variable	Overweight/obesity		p value
	Yes (n=287)	No (n=345)	
Age (years)	43.5 (16.5)	41.5 (17.3)	0.29
Married	133 (46.2)	124 (36.2)	0.25
White colour	70 (24.7)	74 (21.6)	0.46
Unemployed	214 (74.6)	260 (75.5)	0.52
Per capita (US$ 1.00/day)	58 (20.3)	66 (19.2)	0.40
Religious	233 (81.2)	285 (82.7)	0.72
Smoking	49 (17.1)	79 (22.9)	0.39
Drinking every day	20 (7.1)	35 (10.1)	0.34
Short stature	114 (40.0)	97 (28.1)	0.02
Sedentary	46 (16.2)	66 (19.3)	0.54
Abdominal waist >88 cm	189 (66.2)	67 (19.6)	0.00
Energy intake (kcal/day)	2,425±929*	1,904±384*	0.01

*Standard deviation

Figures in parentheses indicate percentages unless otherwise indicated.

**Table 2.** Logistic multiple regression for overweight/obesity in function of short stature, abdominal circumference and energy intake

Characteristics	Overweight/obesity		p value	OR* (95% CI)	OR† (95% CI)	p value
	Yes	No				
Short stature (%)	68 (63.6)	39 (36.4)	0.028	1.7(1.1-2.8)	2.0 (1.1-2.6)	0.02
Abdominal wasting >88 cm, no. (%)	128 (79.5)	33 (20.5)	<0.001	9.8(5.8-16.6)	9.1(5.2-15.9)	<0.001
Energy intake (kcal/day)(mean±SD)	2396.5±40.6	2015.3±86.1	<0.001	1.1‡ (1.1-1.2)	1.1‡ (1.04-1.17)	0.001

*Unadjusted OR;

†Adjusted OR;

‡For an increase of 100 kcal/day;

OR=Odds ratio;

SD=Standard deviation

## DISCUSSION

Despite the low socioeconomic strata of the study population, the prevalence (45%) of overweight and obesity among women was slightly higher than the prevalence (39%) found among adults from all social strata in Brazil's national survey conducted by the Institute of Geography and Statistics (IBGE) (15). As the present study assessed women aged 20-60 years—a group known to be vulnerable to obesity, the prevalence may not be directly comparable with the data of the national survey but the higher proportion of obese women from a Recife slum coincides with the perception that obesity-associated non-communicable diseases are growing faster in the poorer areas of Brazil. Although there are a few comparable studies in urban slums, our results are similar to those found in Bangkok and India (16-18). All these findings seem to confirm the warning of the Expert Consultation on Obesity of the World Health Organization held in 1997 that the epidemic of obesity would reach the poorest regions of the world (19).

Traditional explanations for the increase in obesi-ty include reduced physical activity and consumption of high-energy diets (3,4). We found a very low prevalence (19%) of physical inactivity which was not associated with overweight/obesity. House-keeping activities that demand high-energy expenditure might partly explain our findings as the slum studied had a high demographic density and few spaces to stimulate leisure-time physical activities. Unfortunately, we found no studies that have used the IPAQ for assessing physical activity in a very poor community to compare our results. The relationship among physical activity, obesity, and socioeconomic level is complex and may depend on intrinsic characteristics of the communities.

We found an association between overweight/obesity and high energy intake. The energy intake of the population was studied using 24-hour dietary recall. The foods most consumed by the study population were those of lower cost, usually with high levels of saturated fat or simple carbohydrates. A study in northeast of Brazil with a very low-income population observed that energy intake was below the recommended dietary allowance (about 63%), and stunted obese individuals consumed less energy than the population as a whole (20). Our results did not agree with these findings but in our study, although the community was living at nutritional risk, at the time of the study, many families were receiving a monthly stipend from the Government of Brazil (‘Bolsa Família’), which covered funds for the minimum calorie requirements. Some studies have suggested that the obesity epidemic has occurred despite minimal or no increase in per-capita energy intake and/or energy from the food supply (10,11).

Ours results suggest that the low stature, a reflex of chronic undernutrition in early life, could help explain the increasing prevalence of overweight/obesity in poor communities. These findings are in agreement with those of other studies (10,11). A recent cross-sectional telephone survey with 54,369 Brazilian adults found that a high BMI was strongly associated with short stature (21). Intergenerational consequences of malnutrition and poor health of mothers may lead to impaired phenotypes in their offspring. Also changes in metabolic pathways towards reduced fat oxidation and increased metabolism of carbohydrate may explain this phenomenon, in part.

### Limitations

We assessed energy intake, physical activity, and height at the same time but we did not assess energy expenditure. The use of dietary recall questionnaire for the calculation of calorie intake has been criticized. People under-report their energy intake, and sometimes a higher BMI is associated with a greater degree of under-reporting. However, dietary recall has continued to be employed in most studies that evaluate energy intake. The shorter version of the IPAQ, although addressing the four components of activity, does not allow us to distinguish leisure, occupation, household, or transportation activities, which would be useful for understanding the patterns of behaviour in the slum. Furthermore, present short stature does not necessarily mean malnutrition in early life.

### Conclusions

We found a high prevalence of overweight/obesity associated with high energy intake and short stature in a very poor community. Short stature as a consequence of chronic undernutrition in early life associated with overweight/obesity is in agreement with Barker's hypothesis and helps explain the high prevalence of overweight/obesity in this population. Co-morbidities associated with obesity, especially cardiovascular and metabolic diseases, may cause a greater burden on this population because they have limited access to education and healthcare. For this reason, socially-vulnerable people, such as those living in slums, deserve a higher level of attention by public-health authorities.

## ACKNOWLEDGEMENTS

The study was supported by Brazil's National Council for Scientific and Technological Development (CNPq) and Fundação de Amparo à Ciência e Tecnologia do Estado de Pernambuco.
